# Complete Genome Sequence of the Circulatory Foot-and-Mouth Disease Virus Serotype Asia1 in Bangladesh

**DOI:** 10.1128/genomeA.01135-17

**Published:** 2017-10-26

**Authors:** M. Rahmat Ali, A. S. M. Rubayet Ul Alam, M. Al Amin, Huzzat Ullah, Mohammad Anwar Siddique, Samina Momtaz, Munawar Sultana, M. Anwar Hossain

**Affiliations:** Department of Microbiology, University of Dhaka, Dhaka, Bangladesh

## Abstract

The complete genome sequence of foot-and-mouth disease virus (FMDV) serotype Asia1 isolated from Bangladesh is reported here. Genome analysis revealed amino acid substitutions in the VP1 antigenic region and deletions in both the 5′ and 3′ untranslated regions (UTRs) compared to the genome of the existing vaccine strain (GenBank accession no. AY304994).

## GENOME ANNOUNCEMENT

Foot-and-mouth disease (FMD) is a highly devastating vesicular animal disease of transboundary importance affecting livestock worldwide (1). FMD virus (FMDV), the causative agent, contains a single-stranded, positive-sense RNA and belongs to the *Aphthovirus* genus of the *Picornaviridae* family. Its seven distinct serotypes, namely O, A, Asia1, C, and SAT 1 to 3, are featured with diverse topotypes, genetic lineages, and strains (2). Since 2012, our initiative has been to develop an FMDV seed bank with circulatory serotypes in Bangladesh, and we have reported complete genome sequences of serotypes O and A (3–5). FMDV serotype Asia1 has very low abundance in Bangladesh, but it was first identified serologically in 1996 and our group has reported its reemergence during the 2012–2013 outbreaks (6).

We report here the first complete genome sequence of an FMDV serotype Asia1 isolate, BAN/TA/Ma-167/2013, collected from the tongue epithelium of ruptured vesicles of an infected cow on 23 July 2013 at Madhupur, Tangail, Bangladesh. Virus isolation was performed in the BHK-21 cell line, viral RNA was extracted from the infected cell culture supernatant at passage 3, and cDNA was synthesized with random and oligo(dT) primers. Briefly, 17 overlapping genomic segments were amplified to span the entire viral genome using internal primer pairs, sequenced with an ABI genetic analyzer, and the contigs were assembled into a whole genome using SeqMan version 7.0 (DNAStar Lasergene, USA). Alignment, phylogenetic, and mutational analyses were conducted using MEGA7.

The complete genome of strain BAN/TA/Ma-167/2013 is 8,198 nucleotides (nt) in length and includes a 5′ untranslated region (UTR) of 1,094 nt, a 16-nt poly(C) tract, and a 3′ UTR of 114 nt trailed by a ≥23-nt poly(A) tail. A single 6,990-nt open reading frame (ORF) encodes 2,329 amino acids (aa). The contents of A, U, G, and C are 24.7%, 21.5%, 25.7%, and 28.1%, respectively. The VP1 phylogeny clustered the strain within genetic lineage C, which corroborates our previous report describing the circulation of monotypic FMDV Asia1 in Bangladesh (6).

The nucleotide and deduced amino acid sequence identity of BAN/TA/Ma-167/2013 ranged from 89% to 93% and 95% to 98% compared with whole-genome information and corresponding ORFs for some related FMDV type Asia1 strains wherein IND 101-99 (GenBank accession no. DQ989310) showed maximum identity. Strain BAN/TA/Ma-167/2013 was compared to the currently available vaccine strain (IND 63/72, GenBank accession no. AY304994) in Bangladesh and neighboring countries, which revealed that there are three (T48I, I50T, and S58A) and eight (T138E, Q139T, P140T, T141S, L146M, V148A, N155R, and R156Q) aa substitutions across the B-C loop (residues 40 to 60) and G-H loop (positions 133 to 160) of VP1, respectively. Moreover, 2 nucleotide deletions at the S fragment (positions 73 and 74) and another 2 deletions (positions 8145 and 8171) were detected within the 5′ and 3′ UTRs of the genome.

We report the first complete genome sequence of a local FMDV serotype Asia1 strain circulating in Bangladesh. The results of this study will be helpful in selection of the vaccine candidate of Asia1 serotype in this subcontinent, because vaccine failure with trivalent (type O, A, and Asia1) vaccine has been reported (6).

### Accession number(s).

The complete genome sequence of FMDV serotype Asia1 isolate BAN/TA/Ma-167/2013 has been deposited in the NCBI GenBank database under the accession no. MF782478.
